# Developing a quality of life framework from the perspective of laypeople: a qualitative comparison with the EQ-HWB framework

**DOI:** 10.1007/s11136-025-04038-2

**Published:** 2025-10-03

**Authors:** Guangjie Zhang, Yifan Ding, Zhuxin Mao, Zhihao Yang, Nan Luo, Jan Busschbach

**Affiliations:** 1 Section Medical Psychology, Department of Psychiatry, Erasmus MC, Rotterdam, The Netherlands; 2 Centre for Health Economics Research and Modelling Infectious Diseases, Vaccine and Infectious Disease Institute, University of Antwerp, Antwerp, Belgium; 3 Health Services Management Department, Guizhou Medical University, Guiyang, China; 4 Health Systems and Behavioural Sciences Domain, Saw Swee Hock School of Public Health, National University of Singapore, Singapore, Singapore

**Keywords:** Quality of life, Content validity, EQ-HWB, Qualitative interview

## Abstract

**Introduction:**

The EuroQol health and well-being instrument (EQ-HWB™) measures quality of life (QoL) outcomes in health, public health, and social care settings. Its conceptual framework is rooted in QoL theory, a multidimensional concept encompassing social, psychological, and physical aspects influenced by cultural factors. The content validity of the EQ-HWB remains unexplored in China. This study addressed this gap through qualitative interviews with Chinese laypeople, uncovering their QoL conceptual framework and comparing it with the EQ-HWB’s to evaluate how well it captures its intended outcomes.

**Methods:**

Quota sampling recruited respondents from two regions in China, ensuring diversity in age, gender, education, health conditions, and caregiving experience. Semi-structured qualitative interviews were conducted, transcribed verbatim, and analyzed by two coders using a thematic framework approach. The coders refined codes through consensus, removed irrelevant ones based on set criteria, and organized the remaining codes into sub-themes and themes to develop a Chinese QoL conceptual framework. Lastly, this Chinese QoL framework was compared with the EQ-HWB conceptual framework.

**Results:**

Thirty respondents were recruited and interviewed, achieving data saturation in the last three interviews. From 221 initial codes, 187 were retained to develop a conceptual framework comprising eight themes: feeling and emotion, cognition, self-identity, coping, physical sensation, relationship, activity, and mindset. This framework largely aligned with the EQ-HWB conceptual framework, except for the absence of the ‘mindset’ theme.

**Conclusion:**

The conceptual framework of the EQ-HWB is well-represented within the QoL framework. The findings support the content validity of the EQ-HWB among laypeople in the Chinese context.

**Supplementary Information:**

The online version contains supplementary material available at 10.1007/s11136-025-04038-2.

## Plain English Summary

1. Why is this study needed?
  The content validity of the EQ-HWB has not been explored in Asian populations. Since its conceptual framework is based on Western QoL theory, there is concern that it may not fully capture key aspects of QoL from a Chinese perspective. This gap could compromise its validity in a Chinese context.
2. What is the key problem/issue/question this manuscript addresses?
  This study evaluates the content validity of the EQ-HWB in China. Our findings provide valuable evidence on its relevance in a Chinese context, highlighting its potential for future application.
3. What is the main point of your study?
  A Chinese QoL framework with eight domains was developed. Compared to the EQ-HWB framework, the Chinese framework included all seven common domains and one additional domain, ‘mindset’. This supports the content validity of the EQ-HWB in China.
4. What are your main results and what do they mean?
  A Chinese QoL framework was created and compared it with the EQ-HWB framework, finding alignment between them. While some sub-themes and one theme were missing from the EQ-HWB framework, these omissions are unlikely to impact its content validity.

## Introduction

Outcome measures are commonly used to support decision-making in resource allocation [1]. However, to meet the needs of allocation across both healthcare and social care systems, traditional health-based instruments, such as the EQ-5D-5L and SF-6D, have shown limitations in capturing outcomes relevant to social care [2, 3]. The EuroQol Health and Wellbeing (EQ-HWB) is a new instrument designed to measure intervention outcomes in healthcare, public health, and social care for generic populations, patients, and both formal and informal carers [4–7]. Its conceptual framework is based on the Quality of Life (QoL) framework reported by Wilson and Cleary [4, 8]. The EQ-HWB domains were identified and refined through qualitative reviews of the literature [7, 9]. The item pool was derived from existing questionnaires and item banks commonly used to measure QoL in generic populations, carers, and patients [10]. Items were selected based on psychometric studies [10, 11]. The final EQ-HWB design assesses QoL outcomes using 25 items organized into seven domains: feelings and emotions, cognition, self-identity, coping, physical sensation, relationship, and activity [12, 13].

Previous studies reported that the face validity of EQ-HWB candidate items had been reviewed by the Chinese population [10, 14]. However, the content validity of the EQ-HWB has not yet been comprehensively explored in China. Content validity evaluates how well an instrument captures the targeted aspects it is designed to measure [15]. It is a prerequisite for other forms of validity, such as construct validity and criterion validity [16]. The Consensus-based Standard for the selection of health Measurement Instruments (COSMIN) outlines three criteria for assessing content validity in clinical settings: ‘relevance’, ‘comprehensiveness’, and ‘comprehensibility’. Comprehensiveness ensures that the conceptual framework of the instrument includes all key concepts from respondents’ perspectives [17, 18]. The Food and Drug Administration (FDA) issued a guideline for developing instruments that apply patient-reported outcome measures, stating that evidence of an instrument’s content validity should be appropriate and comprehensive in relation to its intended measurement concept based on qualitative interviews in 2009 [19]. More recently, similar recommendations have been made in the latest guidelines, suggesting that participants should be invited to discuss how they understand and respond to the instructions and items in a PRO measure [20].

The structure of the EQ-HWB was developed from a qualitative literature review, but not from qualitative interviews with targeted populations. Concerns exist about whether the content validity of the EQ-HWB can comprehensively and completely capture all relevant aspects of QoL for its target populations. To date, only one study has examined the content validity of the EQ-HWB using direct input from target groups, such as patients and informal carers [5]. In this study, while the EQ-HWB demonstrated content validity in the Italian population, two aspects of QoL that were not included in the EQ-HWB conceptual framework were identified, namely, living conditions and the possibility of accessing services. Moreover, previous research has argued that the EQ-HWB may not reflect Eastern perspectives on QoL [14], as the EQ-HWB was initially developed based on literature review [9], which was primarily influenced by Western perspectives. Although Chinese stakeholders, like patients and informal caregivers, were involved in the psychometric testing of the EQ-HWB, they did not contribute to the development of the conceptual framework [10, 11]. Therefore, concerns remain that the content validity of the EQ-HWB may be compromised in a Chinese context, since it is unclear whether the instrument accurately reflects Chinese QoL perceptions [21].

Given that cultural factors and experience can shape QoL conceptualizations [22–24], for instance, Shek et al. reported that happiness and satisfaction are import aspects under Western culture, but Chinese culture emphasize forbearance, endurance and humility [25]. Considering differences between Western and Eastern cultures, we hypothesize that the Chinese population’s QoL will differ from the EQ-HWB conceptual framework. These differences may compromise the content validity of the EQ-HWB in China, or they may prove to be insignificant, with limited impact on its application in the Chinese context.

To address these concerns, this study aimed to: (1) develop a conceptual framework of QoL related to health and well-being measurement in China; (2) compare this measurement framework with the EQ-HWB conceptual framework, to examine whether the EQ-HWB reflects such measurement perspective from a Chinese lay perspective. Notably, this article focuses solely on the comprehensiveness aspect of content validity, while the other two aspects, ‘relevance’ and ‘comprehensibility’, will be addressed in a future separate article.

## Methods

Semi-structured, face-to-face qualitative interviews were conducted, following as closely as possible a published protocol from a study exploring the EQ-HWB’s content validity in an Italian population [5], in order to allow for the best possible international comparisons. The study was reported in accordance with the consolidated criteria for reporting qualitative research (COREQ), a 32-item checklist designed to guide researchers in systematically reporting key aspects of qualitative studies [26].

### Research team and reflexivity

The research team comprised six researchers experienced in QoL research and qualitative interviews. GZ, who conducted all interviews, was the only team member with direct participant interaction. GZ refined her interview skills through two pilot rounds with three respondents each. ZY and ZM reviewed each interview transcript during the pilot stage and verified the quality of the interviews at the end of the pilot study. NL initially established five criteria for filtering codes during the analysis stage. All six members reviewed and refined the criteria collectively until consensus was reached, ensuring shared understanding and consistency during the coding process. GZ and YD completed the data analysis and generated an analytical framework. ZY, ZM, and JB reviewed the results and provided feedback on the QoL framework.

In line with the AMEE guidelines, our team held regular meetings to reflect on and discuss reflexivity throughout the project. During the interview stage, ZY and MZ were responsible for overseeing interview quality and monitoring GZ’s techniques. In the data analysis stage, GZ and YD interpreted the nuances in the data and the reasoning behind the creation of codes, which were then discussed collaboratively with ZY and MZ. Weekly meetings were held among GZ, YD, MZ, and ZY to review and refine the development of the conceptual framework. This collaborative process continued until consensus was reached, helping to avoid the influence of GZ and YD’s lived experiences on the coding and framework development.

### Study design

#### Theoretical framework

The study was a qualitative study based on individual semi-structured interviews. It employed thematic analysis, using both inductive and deductive approaches to identify and refine themes from the interview data [27].

#### Participant selection and setting

Using quota sampling, we recruited 30 respondents, ensuring diversity in age, gender, education, medical condition, and registered residence area (rural/urban, known as Hukou). Limits were set for higher education (≤ 12 respondents with university or above) and age (≥ 15 respondents over 45 years). The 30 respondents were divided into three groups: healthy individuals, patients, and informal caregivers, with 10 participants in each group, exceeding the minimum recommended numbers from the COSMIN guideline on assessing content validity [17]. These criteria were established because these three populations are the target users of the EQ-HWB, and perceptions of health and quality of life are known to be influenced by age, education, health conditions, and personal experiences. To comprehensively capture a broader range of QoL perspectives among the Chinese population, we set specific quotas across key demographic and health-related characteristics.

Patients were included only if they had been diagnosed with a health condition. Inclusion criteria required that participants met the sampling requirements and were capable of completing both a semi-structured interview lasting over one hour and the EQ-HWB independently. Recruitment advertisements were posted on community bulletin boards, and interested individuals were screened for eligibility. After agreeing to participate, respondents were invited to a quiet public setting (e.g., a café or park) to complete a semi-structured qualitative interview conducted offline and in person.

#### Data collection

The interviews were conducted using a standard topic guide, which was initially developed in English by QoL measurement experts, then translated into Chinese by GZ and reviewed by ZY and ZM. It was tested during two rounds of pilot interviews with a total of six participants and adjusted based on the results. During the pilot study, respondents found ‘poor well-being’ difficult to understand, as ‘well-being’ usually conveys positive meanings in Chinese culture. Therefore, ‘QoL’ was used instead in the formal interviews. The topic guide included an interviewer self-introduction, research background introduction, warm-up questions, and the QoL topic section. It is provided in Appendix 1.

In the QoL topic section, interviews followed a structured process. Respondents first answered open-ended questions about their perceptions of QoL, then rated their QoL on a 1–10 scale and explained their score. Subsequently, respondents were prompted to identify what poor QoL meant and to provided at least one specific example to illustrate poor QoL. At the end of each interview, the interviewer provided an on-the-spot summary of the participant’s responses and confirmed whether the interpretations accurately reflected their perspectives. Participants were also asked if they had anything to add or clarify. Ethical approval for this study was granted by Guizhou Medical university (2023-03-27). Data collection took place between March and June 2023. All interviews were encrypted audio recorded with participants’ consent for transcription and analysis.

### Analysis

After the face-to-face interviews, each interview was transcribed verbatim in simplified Chinese. Data analysis followed Gale’s thematic analysis methodology [28], involving five steps: (1) familiarizing with the transcripts, (2) coding using inductive and deductive approaches, (3) developing an analytical framework, (4) charting data into a framework matrix, and (5) interpreting the data. The analysis was conducted in Chinese, with careful attention paid to English translations of sub-themes and themes. The total number of codes was reported to reflect the scope of coding across the dataset; however, the frequency of code occurrences was not considered in the analysis. All data were imported and analyzed using NVivo 14.

#### Familiarization and coding

Two coders (GZ and YD) received training in the thematic analysis methodology. They first familiarized themselves with each transcript. Next, they read the transcripts line by line, using codes to extract important information. Both inductive and deductive approaches were applied to generate codes. Deductively, 96 candidate items from the EQ-HWB were used as the initial codebook, reflecting QoL concepts [9]. Inductively, new codes were created for substantive findings beyond the codebook, including: (1) codes that accurately described respondents’ quotations and (2) codes that focused on QoL outcomes rather than events or accidents affecting QoL. For example, if a person was unable to sleep due to anxiety, resulting in a very dark complexion, the code would be ‘sleep problems’ and ‘dark complexion’.

The two researchers independently coded each transcription and held face-to-face meetings after each interview to confirm agreement on code application and interpretation. In cases where disagreements arose, the coders discussed the issues with supervisors ZY and MZ to reach a consensus among the four. Once consensus was achieved, GZ and YD moved on to the next transcription.

#### Developed, applied, and charted codes into a QoL conceptual framework

After analyzing all transcriptions, the codes were exported to an Excel file. The research team developed five criteria to exclude codes unsuitable for describing personal QoL. The rationale behind these five criteria was grounded in the principles of outcome measurement, which emphasize a holistic, individual-centered perspective focused on the current state and self-assessed perceptions of QoL. The filter prioritized subjective QoL assessments over objective ones. First, codes related to morality, death, external events like accidents, income, other economic factors, and lifestyle choices were excluded. Second, codes describing changes in outcomes over time were removed. For example, ‘longevity’ was deleted because it described living, which involves a time factor. Third, codes unable to distinguish between good and poor QoL, such as personality traits, were removed. For instance, the code ‘quiet’ was deleted because it could not specify whether a person had good or poor QoL. Fourth, codes pertaining to the organ, body system, or cellular level, such as the immune system, were excluded in favor of holistic QoL. Fifth, codes reflecting future rather than present QoL outcomes were excluded. For example, ‘health awareness’ was deleted as it referred to improving awareness to benefit future outcomes rather than current QoL.

The QoL framework was developed in three stages. First, codes with similar concepts or keywords, whether positive or negative, were grouped into sub-themes. For example, codes like ‘I feel happy’ and ‘I feel unhappy’ were grouped under ‘feeling of happiness’. Second, sub-themes were formed by combining codes that described similar outcomes. For example, ‘security’ and ‘safety’ were combined into one sub-theme, ‘safety’. Finally, sub-themes were categorized into themes, grouping those with similar attributes, aspects, or closely aligned connotations. For instance, sub-themes such as ‘feeling of happiness’, ‘anxious’, and ‘depressed’, related to feelings or emotions, were grouped under a theme reflecting emotional states. Similarly, sub-themes like ‘mobility’, ‘vision’, and ‘sleeping’ were categorized together, as they describe different abilities or functional aspects of daily living. Additionally, sub-themes with aligned connotations based on respondents’ quotes were grouped accordingly. For example, ‘energetic’ was categorized under ‘physical sensation’, as respondents described it as a state of vitality and physical energy. Since the multidimensionality of QoL is often expressed in terms of different ‘domains’, the ‘theme’ can be considered equivalent to a ‘domain’ of QoL. GZ and YD developed the framework, which was reviewed and finalized by ZY and ZM through consensus.

#### Compare with the EQ-HWB for testing content validity

The analytical framework developed in this study was compared with the EQ-HWB conceptual framework at both the sub-theme and theme levels to identify similarities and differences between the two. This comparison was conducted to assess whether the EQ-HWB adequately captures QoL oncepts as described by Chinese respondents.

## Results

### Participants

A total of 30 respondents were recruited according to our a priori quotas, as outlined in the demographic information presented in Table 1.

**Table 1:** Sample characteristics

Respondent	Sex	Age	Registered residence area	Group	Education
P1	Male	23	Urban Hukou	Informal caregiver	High education
P2	Female	54	Urban Hukou	Informal caregiver	Low education
P3	Male	35	Rural Hukou	Patient	Low education
P4	Female	65	Urban Hukou	Informal caregiver	Low education
P5	Male	51	Urban Hukou	Patient	Low education
P6	Female	55	Urban Hukou	Informal caregiver	Low education
P7	Female	58	Urban Hukou	Informal caregiver	Low education
P8	Female	64	Rural Hukou	Patient	Low education
P9	Male	60	Rural Hukou	Patient	Low education
P10	Male	53	Urban Hukou	Informal caregiver	High education
P11	Male	42	Rural Hukou	Patient	Low education
P12	Female	58	Urban Hukou	Patient	Low education
P13	Male	55	Urban Hukou	Patient	Low education
P14	Female	27	Urban Hukou	Healthy	High education
P15	Male	23	Urban Hukou	Healthy	High education
P16	Female	26	Rural Hukou	Healthy	High education
P17	Female	45	Urban Hukou	Informal caregiver	High education
P18	Female	38	Urban Hukou	Healthy	High education
P19	Male	29	Urban Hukou	Healthy	High education
P20	Female	26	Rural Hukou	Healthy	High education
P21	Male	20	Rural Hukou	Healthy	High education
P22	Male	55	Rural Hukou	Informal caregiver	Low education
P23	Female	53	Urban Hukou	Patient	Low education
P24	Male	18	Urban Hukou	Healthy	Low education
P25	Female	18	Urban Hukou	Informal caregiver	Low education
P26	Male	18	Rural Hukou	Informal caregiver	Low education
P27	Female	51	Rural Hukou	Patient	Low education
P28	Male	56	Rural Hukou	Patient	Low education
P29	Female	63	Urban Hukou	Healthy	Low education
P30	Male	18	Rural Hukou	Healthy	Low education

Urban Hukou refers to household registration in urban areas; Rural Hukou refers to household registration in rural areas; High education are university and above university; Female (*n* = 15); Age ≤ 45 (*n* = 15); Healthy (*n* = 10); Patient (*n* = 10); Informal caregiver (*n* = 10); Respondents from Guangzhou (*n* = 22); Respondents from Harbin (*n* = 8); Urban Hukou (*n* = 18); High education (*n* = 10)

### QoL framework

A total of 221 codes were generated to summarize QoL-related comments, of which 34 were excluded from the development of the QoL framework based on our predefined criteria. These excluded codes included external impacts (*n* = 11), time-dependent outcomes (*n* = 3), indistinguishable QoL outcomes (*n* = 13), codes pertaining to organs, body systems, or the cellular level (*n* = 5), and codes reflecting future outcomes (*n* = 2). Of the 187 remaining codes, over half (*n* = 97) were developed inductively. We categorized the 187 codes into 57 sub-themes, grouped into eight themes: feeling and emotion, cognition, self-identity, coping, physical sensation, relationship, activity, and mindset. Below, we describe the sub-themes and themes in our analytical framework, focusing on the new findings compared to the EQ-HWB conceptual framework, as shown in Table 2. The full quotes selected for each sub-theme presented in the manuscript are provided in Table 3. Additional Appendix 2 has been provided to present all sub-themes and representative quotes mentioned in the QoL framework.

**Table 2:** Comparison between the analytical framework and conceptual framework of the EQ-HWB

Theme	Sub-theme				
Feeling and emotion	Worry	Overwhelmed	Fear	Anxious	Relax and calm
	Depressed and frustrated	Sad	Satisfaction	Feeling of Happiness	Safety
	Anger	Regret	Expectation	Hopeless	Boring
	Stressful	Emotion related ability			
Cognition	Confused	Thinking clearly	Concentrate	Memory	Cognitive impairment
Self-identity	Dignitified/Disrespected	Self-assessment	Self-perception		
Coping	Cope	Reliance	Adaption	Control	
Relationship	Betrayal	Support	Get on with people	Socialization	Judgment
	Sense of belonging	Loneliness	Burden	Interpersonal relationship	
Physical sensation	Exhausted	Energetic	Discomfort	Pain	Weakness
	Appetite	Appearance			
Activity	Enjoyable or meaningful activities/role	physical function	Daily activity	Self-care	Diet-what to eat and how to eat
	Mobility	Vision	Verbal expression	Sleep	Hearing
Mindset	Life attitude	Adjust mindset	Negative/positive energy		

Cells highlighted in yellow were new findings found from our QoL conceptual framework; cells highlighted in green were found in the EQ-HWB but were missing from our QoL conceptual framework

**Table 3:** Selected respondents’ quote

Theme	Sub-theme	Explanation	Quote
Feeling and emotion	Regret	‘Regret’, a negative emotion, arises when the development of events or situations differs from what was expected after a decision is made	*“When she first discovered she had cancer, it was only the size of a soybean. She said she relied on traditional Chinese medicine and conservative treatments, but the condition eventually worsened. She also underwent chemotherapy, which initially helped, but the cancer later deteriorated again. Now, the cancer cells have spread to her bone marrow, causing pain in various joints. She deeply regrets this and told me that she should have listened to Western medicine. She said she should have started treatment as soon as she found out. She regrets missing the best time for treatment.” (P6)*
	Boredom	‘Boredom’ referred to a feeling caused by a lack of engagement in interesting or meaningful activities	*“Not being able to go out affects my mood and mental well-being. Being alone at home feels stifling, making me feel very lonely and bored. Sometimes, it feels worse than death; he often says this kind of torment is unbearable and that it would be better to die than to endure prolonged suffering.” (p23)*
	Stress	‘Stress’ was associated with difficulties or dilemmas related to family, academic study, work, relationships, or economic situations. This distress could cause behavioral changes, leading to health problems	*“You have to take care of your own parents while also looking after your children, so the stress is quite significant.”(p2)*
	Emotional abilities	‘Emotional abilities’ referred to the capacity to control, manage, and express emotions, leading to emotional stability and enhanced rational thinking. A person who can effectively control their emotions is likely to maintain emotional stability	*“His emotions are very volatile, suddenly feeling sad, suddenly irritable, suddenly happy, highly fluctuating, and extremely unstable.”(p24)*
Cognition	Cognitive impairment	‘Cognitive impairment’ referred to delays in intellectual development. It may manifest as deficits or reduced abilities in learning, understanding, thinking, memory, and problem-solving	*“This example is from our village. Look, now he’s in his thirties or forties, but he behaves like a child. No matter how you deceive him outside, he just smiles and doesn’t take it seriously. Actually, it’s just that his mind isn’t sharp enough. That’s why he didn’t study and lacks education. Whatever you say, he just accepts it. He wanders everywhere, and when he’s hungry, if someone gives him food, he eats it. If no one feeds him, he goes back home. And even if he goes home, he knows the way back.” (p28)*
Self-identity	Self-assessment	‘Self-assessment’ included both positive and negative aspects of self-evaluation. For example, statements such as: ‘I feel disappointed with myself’, ‘I lack confidence’, and ‘self-denial’ reflected negative self-assessments	*“For example, frequently engaging in self-denial and using various things to invalidate oneself can lead to internal exhaustion.” (p21)* *“A form of spontaneous self-reflection, as Confucius mentioned, involves daily self-examination. This type of introspection means that when I face setbacks or failures, my initial thought is not "Why did I fail?" but rather "What caused my failure?" I focus on the reasons behind the failure instead of the failure itself, identifying my own shortcomings.” (P26)*
Coping	Adaptation	‘Adaptation’ refers to the process by which people adjust to external environments and distress	*“Previously, he didn’t drink much and rarely smoked. However, after one of his children had an accident and passed away, it seemed like the impact was too overwhelming for him. He started smoking and drinking frequently.” (p3)* *“Peers may experience lower quality of life in terms of mental well-being due to academic pressure. They may suffer from depression, become irritable, and have poor self-regulation and might not adapt well to the outside world.” (p24)*
Physical sensation	Weakness	‘Weakness’ describes a state of physical or mental feebleness, such as experiencing reduced bodily functions after surgery, making walking feel strenuous	*“Before I had the stent placed, I was quite energetic. I could easily climb stairs, and I could even jog up to the 5th floor. I used to live on the 5th floor. Now, after the surgery, I feel weak and lack energy. Even climbing stairs now leaves me short of breath.” (p28)*
	Appetite	‘Appetite’ refers to a decrease in appetite or a lack of desire to eat caused by disease or medical treatment	*“For example, they don’t want to eat, even if you give them food, and even if you give them food, they still won’t eat.” (p22)*
	Appearance	‘Appearance’ referred to changes in appearance caused by disease, such as a darkened complexion. It also included spiritual appearance, reflecting a person’s positive, vibrant, and energetic state as expressed through their outward appearance	*“Difficulty falling asleep and a very dark complexion.” (p19)* *“Perhaps there could still be some impact on his mental outlook. For instance, before he started relying on his parents, when he was in his twenties, he might have been very ambitious and had a youthful vigor and vitality that gave the impression of a young man full of energy. Of course, after he began relying on his parents and didn’t actively seek work, he might become lazy and lose some of that energy. Gradually, he may lose some of the spirit and vitality that young people typically have.” (p21)*
Relationship	Establishing relationships	‘Establishing relationships’ refers to the process of forming and nurturing connections with others, such as workplace relationships	*You definitely need to gain recognition from your boss. When you do something, you must focus and be serious about it. I make sure my work doesn’t trouble my boss. If you do your job well, those above you won’t have to worry about it. But if you don’t do it properly, if there are mistakes here and there, I won’t let them criticize me, understand? Only by doing this will it work. (p3)*
	Judgment	‘Judgment’ was created to capture feelings of being judged, discussed, or discriminated against	*“For example, others may discriminate against you, including those close to you who were once very supportive but now, over time, have also started to discriminate against you.” (p17)*
	Betrayal	‘Betrayal’ refers to a personal feeling of broken trust or a violated relationship, negatively impacting an individual’s QoL	*“However, after that person exploited him, he started a new phase of life. He felt betrayed and hurt, having invested a lot of money, time, and energy, but he gained nothing in return.” (p15)*
Activity	Physical function	The ‘Physical function’ sub-theme was created to express higher physical capabilities than mobility, such as going to the gym	*“For example, activities like practicing Tai Chi, dancing, and singing are more enjoyable and preferable.” (p29)*
	Sleep	‘Sleep’ was aligned with this theme because it describes the ability to fall asleep or stay asleep	*“After watching TV and using the phone for too long, sometimes I feel very tired but can’t sleep, which affects my ability to fall asleep.” (p29)*
	Diet	‘Diet’ was introduced as a new sub-theme representing the ability to eat food	*“He just feels nauseous when he sees us eating meat; he’s quite peculiar. He only eats fish and eggs.” (p27)*
Mindset	Life attitude	‘Life attitude’ referred to the attitude towards life	*“Quality of life means that your life is your own, and you need to value it. You should not treat your life as a joke.” (p9)*
	Adjust mindset	‘Adjust mindset’ was adjusting one’s way of thinking about difficulties or challenges when they arise	*“Depending on the severity of the illness, if the disease is severe and beyond one’s ability to cope, they are likely to feel very pessimistic and despondent.” (p10)* *“After falling ill, my mood definitely affects my condition. However, once I let go and stop worrying about other things, everything seems fine. Once I change my mindset, I no longer mind. For example, when I initially felt unwell, I found fault with everything my husband cooked. But later, when I changed my perspective, it didn’t bother me anymore.” (p12)*
	Positive/negative energy	‘Positive/negative energy’ was influencing those around them by conveying personal emotions or opinions	*“He tends to blame others, attributing his own miserable life to his relatives and siblings. He believes it’s their fault for not helping him. He feels they should assist him with his tasks but don’t, and as a result, he often blames others and sometimes even resorts to scolding them.” (p15)*

#### Feeling and emotion

Feeling and emotion encompassed various positive and negative emotions, which were affected by events, diseases, or accidents. This theme contained the most sub-themes in either the Chinese QoL framework or the EQ-HWB framework. It included 18 sub-themes, with four new sub-themes not present in the EQ-HWB framework: ‘Regret’, ‘Boredom’, ‘Stress’, and ‘Emotional abilities’.

**Regret**

‘Regret’, a negative emotion, arises when the development of events or situations differs from what was expected after a decision is made.

“When she first discovered she had cancer…… She deeply regrets this and……She regrets missing……” (P6)

**Stress**

‘Stress’ was associated with difficulties or dilemmas related to family, academic study, work, relationships, or economic situations. This distress could cause behavioral changes, leading to health problems.

“You have to take care of your own parents while also looking after your children, so the stress is quite significant.”(p2)

**Boredom**

‘Boredom’ referred to a feeling caused by a lack of engagement in interesting or meaningful activities.

“Being alone at home feels stifling, making me feel very lonely and bored.” (p23)

**Emotional abilities**

‘Emotional abilities’ referred to the capacity to control, manage, and express emotions, leading to emotional stability and enhanced rational thinking. A person who can effectively control their emotions is likely to maintain emotional stability.

“His emotions are very volatile, suddenly feeling sad, suddenly irritable, suddenly happy, highly fluctuating, and extremely unstable.”(p24)

#### Cognition

The ‘cognition’ theme aligned with the EQ-HWB framework, sharing the same sub-themes: confusion, clear thinking, concentration, and memory. An additional sub-theme, ‘cognitive impairment’, was identified.

**Cognitive impairment**

‘Cognitive impairment’ referred to delays in intellectual development. It may manifest as deficits or reduced abilities in learning, understanding, thinking, memory, and problem-solving.

“He is in his thirties or forties, but he behaves like a child……he just smiles and doesn’t take it seriously……” (p28)

#### Self-identity

The ‘self-identity’ theme encompassed three sub-themes: dignified/disrespected, self-perception, and an additional sub-theme, ‘self-assessment’.

**Self-assessment**

‘Self-assessment’ included both positive and negative aspects of self-evaluation. For example, statements such as: ‘I feel disappointed with myself’, ‘I lack confidence’, and ‘self-denial’ reflected negative self-assessments.

“……frequently engaging in self-denial and using various things to invalidate oneself can lead to internal exhaustion.” (p21)

“A form of spontaneous self-reflection…… identifying my own shortcomings.” (P26)

#### Coping

‘Coping’ is defined as the ability to cope with and adapt to changes in external environments, including natural and social environments. This theme contained four sub-themes, with an additional sub-theme—‘adaptation’—identified.

**Adaptation**

‘Adaptation’ refers to the process by which people adjust to external environments and distress.

“Previously……However, after one of his children had an accident and passed away……started smoking and drinking frequently.” (p3)

“Peers might have depression……and might not adapt well to the outside world.” (p24)

#### Physical sensation

Physical sensation was another significant theme, sharing similar concepts with the EQ-HWB framework and including six sub-themes. Three additional sub-themes—weakness, appetite, and appearance—were identified in our framework. These new sub-themes described detailed physical sensations impacted by illness or medical treatment.

**Weakness**

‘Weakness’ describes a state of physical or mental feebleness, such as experiencing reduced bodily functions after surgery, making walking feel strenuous.

“Before I had the stent placed…… I feel weak and lack energy. Even climbing stairs now leaves me short of breath.” (p28)

**Appetite**

‘Appetite’ refers to a decrease in appetite or a lack of desire to eat caused by disease or medical treatment.

“…… they don’t want to eat, and even if you give them food, they still won’t eat.”(p22)

**Appearance**

‘Appearance’ referred to changes in appearance caused by disease, such as a darkened complexion. It also included spiritual appearance, reflecting a person’s positive, vibrant, and energetic state as expressed through their outward appearance.

“Difficulty falling asleep and a very dark complexion.” (p19)

“……some impact on his mental outlook. ….. become lazy and lose some of that energy. Gradually, he may lose some of the spirit and vitality……” (p21)

#### Relationship

‘Relationship’ was an important aspect of QoL and was frequently mentioned by respondents. This theme shared similar content with the EQ-HWB framework but included three newly identified sub-themes: ‘establishing relationships’, ‘judgment’, and ‘betrayal’.

**Establishing relationships**

‘Establishing relationships’ refers to the process of forming and nurturing connections with others, such as workplace relationships.

“I make sure my work doesn’t trouble my boss……only by doing this will it work.”(p3)

**Judgment**

‘Judgment’ was created to capture feelings of being judged, discussed, or discriminated against.

“For example, others may discriminate against you……” (p17)

**Betrayal**

‘Betrayal’ refers to a personal feeling of broken trust or a violated relationship, negatively impacting an individual’s QoL.

“…… he started a new phase of life. He felt betrayed and hurt……” (p15)

#### Activity

The ‘Activity’ theme illustrated people’s capacity to perform basic functions, such as eating, sleeping, walking, and speaking. This theme differs from the EQ-HWB framework, which describes a broader range of activities, including ‘physical function’, ‘sleep’, and ‘diet’.

**Physical function**

The ‘Physical function’ sub-theme was created to express higher physical capabilities than mobility, such as going to the gym.

“For example, activities like practicing Tai Chi, dancing, and singing……” (p29)

**Sleep**

‘Sleep’ was aligned with this theme because it describes the ability to fall asleep or stay asleep.

“……sometimes I feel very tired but can’t sleep, which affects my ability to fall asleep.” (p29)

**Diet**

‘Diet’ was introduced as a new sub-theme representing the ability to eat food.

“……he’s quite peculiar. He only eats fish and eggs.” (p27)

#### Mindset

‘Mindset’ is a newly identified theme for describing QoL and was frequently referenced by respondents. This theme refers to a person’s overall way of viewing and approaching things, including their attitudes and perspectives, ultimately achieving a harmonious and peaceful state that unites the interior and exterior aspects of the self. It encompasses three aspects: first, the attitude towards life (life attitude); second, adjusting one’s way of thinking about difficulties or challenges when they arise (adjust mindset); and third, influencing those around them by conveying personal emotions or opinions (positive/negative energy).

“Quality of life means that your life…… You should not treat your life as a joke.” (p9)

“…… if the disease is severe and beyond one’s ability to cope, they are likely to feel very pessimistic and despondent.” (p10)

“Once I change my mindset, I no longer mind……when I changed my perspective, it didn’t bother me anymore.” (p12)

“He tends to blame others……he often blames others and sometimes even resorts to scolding them.” (p15)

### Comparison with the EQ-HWB

The two frameworks maintain high consistency in general. The Chinese QoL framework demonstrates a broader scope in structure and content compared to the EQ-HWB conceptual framework. The two frameworks also exhibited significant similarity, with alignment rates of 68% (18/57) for sub-themes and 88% (7/8) for themes.

However, some differences were observed between the two frameworks. First, two sub-themes shared the same concepts but were aligned differently: the sub-theme ‘boredom’ fell into the ‘feeling and emotion’ theme in our QoL framework but was part of ‘activity’ in the EQ-HWB framework. Similarly, the sub-theme ‘sleep’ was included in the ‘activity’ theme in our framework but grouped under ‘physical sensation’ in the EQ-HWB framework. Second, we identified a similar sub-theme, ‘autonomy’, as in the original framework, but with broader connotations in our study. Originally, autonomy had a positive meaning, referring to making decisions about one’s life even when relying on others, such as doctors. In contrast, our findings also included a negative aspect, ‘dependence’, which involved relying on other people’s help, ideas, and thoughts, indicating a lack of autonomy in decision-making and independent thinking.

## Discussion

In this study, the comprehensiveness aspect of the EQ-HWB’s content validity was explored. A broader QoL framework than the original EQ-HWB conceptual framework was found, which was almost entirely reflected in our QoL framework, except for the ‘hearing’ sub-theme. It was omitted by our respondents. Both frameworks collectively describe the concept of QoL as encompassing three main factors: physical, psychological, and social.

The new theme, ‘mindset’, was identified, meaning a way of thinking—reflecting a mental framework that shapes how we perceive, interpret, and respond to experiences [29]. It is related to how individuals respond to success and failure and impacts their life direction and sense of life’s worth [30–32]. Huang’s study showed that a positive mindset correlates with higher life satisfaction and happiness and fewer symptoms like poor sleep, appetite loss, and depression [31], highlighting its closer association with QoL than with health or social care [33–35]. Moreover, ‘mindset’ reflects how traditional Chinese philosophies and religions, including Confucianism and Daoism, significantly shape Chinese perspectives on health, happiness, and life [36, 37]. Confucianism views happiness as stemming from life satisfaction and social contributions, while Daoism links it to appreciating nature and life, fostering inner peace and harmony [37, 38]. The theme ‘mindset’ in our framework incorporates aspects of Confucianism and Daoism, as reflected in the sub-themes ‘Life Attitude’, ‘Adjusting Mindset’, and ‘Positive/Negative Energy’. Ding’s study also reported that these traditional factors influence Chinese populations’ views on health-related quality of life [39].

‘Coping’ refers to the practical actions or strategies an individual uses to manage challenges, emphasizing behavioral responses and problem-solving skills. In contrast, ‘mindset’ reflects a cognitive and emotional orientation—how a person interprets or frames a problem—highlighting perspective rather than action. Although the sub-theme ‘adjusting mindset’ may appear similar to coping, it specifically involves internally altering one’s perspective on a problem and emphasizes mental and emotional regulation. This distinction aligns with Western concepts of ‘positive coping strategies’, which include cognitive reframing and self-regulation techniques [40–43]. This may explain why Chinese respondents tend to describe their QoL in terms of mental and emotional regulation and acknowledge the impact of emotional states on physical health [39]. This also further clarifies why ‘mindset’ emerged as a distinct theme in our framework.

Sub-themes such as ‘emotion control’ [44, 45], ‘appetite’ [39, 44, 45], ‘adaptation (social/environment)’ [39, 44, 45], ‘sleep [46]’, ‘spiritual appearance’ [39, 46], and ‘social interaction’ [47] are commonly identified by Chinese researchers but overlooked by Western-based QoL instruments. We found that Chinese laypeople use these outcomes to describe their QoL. However, these newly identified sub-themes do not create new themes, such as ‘mindset’, but instead serve as supplements to the existing themes within the QoL framework. This highlights that, when describing QoL, Chinese individuals consider not only personal aspects but also external environmental factors and their relationships with others. Such an approach reflects how the Chinese population perceives QoL holistically [39, 44, 45, 48].

The first study on the content validity of the EQ-HWB reported that Italian respondents viewed QoL as including ‘the presence or absence of diseases’, ‘the ability to perform activities’, ‘social participation’, ‘emotional functioning’, ‘living conditions’, and ‘the possibility of accessing services’. Although QoL is often seen as culturally grounded [44, 45], our findings did not reveal significant distinctions from the Italian study [5].

More importantly, the QoL conceptual framework from Chinese laypeople aligns not only with the EQ-HWB framework but also with other Western QoL frameworks proposed by scholars [49, 50]. For instance, the WHOQOL assesses QoL as “an individual’s perception of their position in life in the context of the culture in which they live and in relation to their goals, expectations, standards, and concerns” [50]. While this definition does not break down into specific themes or sub-themes—something typically seen when QoL is operationalized into a questionnaire—it captures both subjective and objective dimensions of QoL. This dual perspective, encompassing both individual and social aspects, aligns with our findings [41, 51, 52]. Several researchers, like Moon et al. [53]. or Schalock et al. [54] promoted several specific indicators for assessing QoL. These indicators include: (1) emotional well-being; (2) interpersonal relations; (3) material well-being; (4) personal development; (5) physical well-being; (6) self-determination; (7) social inclusion; and (8) rights [54]. Our QoL framework aligns well with these criteria, except for ‘rights’ and ‘material well-being’. While respondents mentioned aspects of ‘material well-being’, such as economic situation, living conditions, and natural environments, during the interviews, these were not included in our framework due to our criteria, which excluded external factors to focus solely on QoL outcomes.

Both the QoL and EQ-HWB frameworks incorporate physical, mental, and social aspects of QoL. Although the two frameworks demonstrate similar core concepts across Western and Eastern populations, differences do exist. These differences are evident in the usage and phrasing of concepts and should be considered when translating and culturally adapting the EQ-HWB. For instance, the sub-theme ‘sleep’ is categorized under the ‘activity’ theme in the QoL framework because respondents emphasized their ability to fall asleep. In contrast, the EQ-HWB framework associates ‘sleep’ with ‘energetic’ in the ‘physical sensations’ theme, which places more emphasis on the outcome of poor sleep. Additionally, ‘cope with’ is commonly associated with distress, challenges, or problems that people actively fight against, rather than with ‘coping with daily activity’, as used in Western culture. These differences may help explain why the EQ-HWB factor structure does not fit well within the Chinese population, indicating a lack of good model fit [11]. More importantly, these differences may require further efforts in translating and adapting the EQ-HWB for use in China. Similarly, two sub-themes identified in this study—positive energy and negative energy—illustrate translation challenges. In the Chinese context, positive energy refers to optimistic, uplifting emotions, attitudes, and behaviors that promote confidence, motivation, and social harmony, while negative energy describes pessimistic, discouraging emotions and behaviors that spread anxiety, frustration, or negativity to others. Although these expressions were frequently mentioned by respondents, they lack clearly defined or directly equivalent connotations in English. As such, these terms were retained through character-based translation to preserve their original cultural meanings as expressed by the participants.

The EQ-HWB overlooks some aspects of QoL from both Western and Chinese perspectives; however, these omissions are not necessarily significant losses. When developing a QoL instrument, a trade-off in deciding the number of items and dimensions must be considered [55]. For example, ‘life attitude’ or ‘regret’ may already be adequately addressed by other sub-themes such as ‘enjoyable or meaningful activities/role’ and ‘cope’.

## Limitation

This study is not without limitations. The first limitation concerns the diversity of the sample. The respondents from Harbin were young, relatively healthy, and highly educated individuals. Although the requirements of the quota sampling method were met, the inclusion of patients and elderly participants from Harbin could have provided a broader understanding of their perspectives on QoL. Second, as all patients recruited in this study were from community settings and most had chronic diseases, we suggest that future research consider including hospitalized patients with severe illnesses, particularly those who face significant challenges living independently in the community. Third, although the translation of sub-themes and themes was carefully considered, some sub-themes and one theme were difficult to translate accurately. For example, the sub-theme 正/负能量 was translated into ‘Positive/Negative Energy,’ and the theme ‘心态’ was translated into ‘Mindset.’ Moreover, we did not set any criteria for determining data saturation, but we recruited more respondents than the COSMIN recommendation, and similar views on QoL were repeated by the last 10 interviewees. Nevertheless, the final limitation is that we could only determine that the data was saturated subjectively.

## Conclusion

The conceptual framework of the EQ-HWB is well-represented within the QoL framework put forward by laypeople in China. This provides supportive evidence for the comprehensiveness of the instrument, a necessary condition for its validity. Although we identified some missing aspects of QoL, these omissions are unlikely to impact the effectiveness of the EQ-HWB. Overall, the study provides evidence supporting the content validity of the EQ-HWB within the Chinese context.

## Supplementary Information

Below is the link to the electronic supplementary material.

Supplementary file1 (DOCX 27 KB)

## Data Availability

The qualitative data supporting the findings of this study are not publicly available due to privacy concerns. However, anonymised excerpts may be requested from the corresponding author.
