# Supplementary material for: Developing a quality of life framework from the perspective of laypeople: a qualitative comparison with the EQ-HWB framework
Source: Qual Life Res. 2025 Oct 3;34(12):3525–39. doi: 10.1007/s11136-025-04038-2 (PMC12689660; doi:10.1007/s11136-025-04038-2)
Supplement: Supplementary file 1 — Supplementary file1 (DOCX 27 KB) [file 11136_2025_4038_MOESM1_ESM.docx]

# Feeling and emotion

Worry

It's because your parents are getting older, isn't it? You have to worry about them, don't you? Because your parents are at home and you're working alone, right? It's always a worry in your heart. (p11)

Overwhelmed

P: Yes, yes, he can't do it, a lot of things he can't reach anymore, they don't dare to imagine it, they can't imagine it, and then they make a comparison with before they can act before, he will feel that he's far less than he was before then he'll form a kind of vicious circle, and the more he thinks about it, the worse it gets, the worse he gets mentally, and he'll reach such a point. (p10)

Fear

For example, some people will have some fear for some social communication, he may he may make some intention in his daily life against his real will. For example, if you eat in a restaurant, you may be afraid to ask the waiter for help because of some psychological pressure, this is a positive example (p19)

Anxious

If his weight goes up and then the aesthetics of everyone now is on the thinner side and then when he sees some of some of the thinner some of the people on the internet he might feel some anxiety but he but it's hard for him to act on it because he's so used to this diet and then he'll get anxious again and then it'll be this cycle of a very bad thing. (p25)

Relax and calm

I feel that every day I feel quite relaxed, the mood is quite that, sometimes occasionally friends get together to drink a little drink, sometimes feel like chatting, that is to say gossip, mutual encouragement (p3)

Depressed and frustrate

His mentality is probably I think so, and then there are some patients, he is in the process of treatment, he will wear off his own heart is that those toughness, anyway, for example, he will be more positive to cooperate with the treatment in the beginning, but his treatment process, he because he needs to come to the hospital again and again, he may slowly feel that I don't want to come, I'll be like this, I don't want to be cured either (p16)

Sad

Sometimes when we see him say that, we feel sad in our hearts because he is paralyzed, he can't walk or move, but he relies on us to turn him over, and if you bathe him for a long time, he scolds us because he thinks it's okay to just wipe him up, so he doesn't need to be washed so clean. (P2)

Satisfaction

Anyway, I do feel that my own life and work I'm relatively satisfied most of the time, there is nothing that I can particularly complain or what or spit point, I just feel that overall you although that is very plain, but there is nothing that makes me particularly say particularly difficult, or particularly want to what is called what particularly want to spit point, I just feel that overall it is relatively satisfied (P16)

Happy

Specifically according to the reasoning then it should be that if you go to go dancing with people every day, you must be happier, in a better mood, and won't think about those unhappy things at home. (P7)

Safety

Then there is a sense of security, that's all that matters, right? You now and no money, and no house, you say a lot of things that would not have a sense of security, that's the reasoning. Is not it? If a woman wants to follow you, she also wants your sense of security, right? It is not possible that you are always renting a house, is not it, to take a woman to live, right? That's what it is. (p9)

Anger

Like that kind of like that kind of cranky feeling? I don't know which sentence I'm going to say, I'll get all worked up, I'll get all hot-eyed, and then I'll start cursing. Yeah, it's a manifestation of that kind. (p15)

Regret

His regret was told to me, he said he still needs to listen to western medicine, he had to treat him when he found out at that time, he said he has missed it, he regrets that he just missed the best time to treat him. (p6)

Expectation

Nowadays, if the family is not happy, if the two in-laws are always quarreling, some people don't have much hope in life, always quarrelling, and some people even commit suicide. That's why I think it's important to have peace in a family. (p23)

Hopeless

Yes, now this thing he's bedridden, he doesn't have any hope left and feels that he'll be very pessimistic about the future (p20)

Boredom

P: Yeah, he's just very bored lying around, which means that he might be thirsty and wanting to see something in the outside world, but not really. (p17)

Ability with emotion

His moods can be very volatile, suddenly sad, suddenly cranky, suddenly happy, very volatile (p24)

# Cognition

Confused

Feelings may be usually some time may have some confusion, some time may have some confusion when studying, don't know where to start, or a kind of anticipation of the future, or uncertainty of speculation. Anyway, it's just worrying. (p21)

thinking clearly

And then it affects your cognitive problems, such as your inability to think clearly, or your inability to memorize or your ability to concentrate, which causes you to feel that you are not able to continue learning. (P5)

Concentrate

When you are in a singing mood throw away all your worries and sing with a single mind, and when you play Tai Chi let yourself be calm and unhurried. (p29)

Memory

For these things he is brain infarction, that brain blockage, some blood vessels, forgetting a lot of things like that. (P13)

Cognitive impairment

This example should be our village, but you see now he is also thirty or forty years old, as if he is also like a child, casually how you fool him outside, but also smiling, did not take it seriously, in fact, he is just not enough brains, so that also did not read, you see him so that also has no culture, and do not read, you know what to say what it is, and he is also to go wandering around and hungry, of course, you have what He is also wandering around, hungry, of course, you have what to give him to eat to eat, no one to give him food to go home. If go home he still have to go back to recognize go back. (p28)

# Self-identify

I felt like I lived with dignity, don’t feel respected

He just sleeps at night when he sleeps, usually pulls on the bed to pull two or three times to urinate, that is, to make the quilt what mattress is wet, get up in the morning on the bed side to pull urine, that is, the ground floor of the underground bed are all urine, his wife went to work at 7:00 p.m., to be more than 6:00 p.m. to come back (p13)

self-worth

Just because he doesn't exercise or do anything, his energy will be spent on other things. For example, often he will deny himself, often take something to deny himself to just internalize himself. (p21)

Self-perception

Similar to the phrase, I collide with the world as a way of identifying my shape, for him to be a better perception of himself, or whether it's good or bad, and he succeeds he will also be in the midst of failures, and then he will improve himself, and then he will know his own strengths and weaknesses, and all of this presupposes he's in a situation where other people will be supportive of him, and then he's in a situation where he has the material conditions to be able to support him to the very end (p1)

# Coping

Cope

An example of a lower quality of life? I think it seems like. It's that we have a woman here. His family had a good life before, she and her husband divorced, divorced, aunty is about the same age as me, and then he can not stand the blow, can not stand the blow, then slowly their brains are a little bit not very clear, he went to the garbage cans to pick up things to eat. We sometimes see him very poor, we sometimes those bread water or other put him there to give him to eat, he also said thank you, you so good, I said I should, I said if you do not have money, I can also give you a little bit, he said no I have to eat are useful, he said as if they have worn those clothes, but also do not think that he is more appropriate to wear, then you can give him a few pieces of clothing to him to wear. I think it's too much for him to do this kind of thing. I think it's not right for him, you see, he's a middle-aged man, he has a son and a daughter, and after his husband divorced him, he couldn't accept the fact that he made himself miserable, as if he didn't have any ambition, and as if he was in a trance, as if he wasn't very normal anymore. I have seen them go to the garbage can to pick up something to eat, I said why don't you go home, he said I don't even want to go home. I said, "Do you have a place to stay? (P2)

Adaption

A good state of mind is to be quite happy every day, there's nothing to worry about, but it's not that you can't meet it, it's just that if you meet it you've got to get across it anyway, and anyway, you feel that if you meet it then it's quite difficult, and then it's bothering you, but if you if you're just happy or not, he's all there, and then why not be a little bit happier? Right? (P18)

Control

I cook for myself when I am able to move, even if I am not feeling well myself, I cook for myself when I am able to cook, and first of all there is this feeling that if I cook like this knowing that I can't eat for myself, do you want me to die. These are unavoidable, dietary aspects (p12)

Reliance

He has to rely on others for everything. He can't do anything else except eat with chopsticks and stand up and walk a couple of steps, and that's how far he's gotten. (P10)

# Physical sensation

Exhausted

He doesn't say it, but we get the sense that what he doesn't say is verbal speech, because maybe the strength isn't there anymore either, and now the speech is speaking less after all. That's one of the obvious differences, right there. His language is less, the content of his speech is less, and his mental state is worse, which is a very obvious sign, but of course it has something to do with the weakening of his body, which continues to be weakened, and it's all caused by various aspects of this condition. His mental condition, he's definitely not well (P10)

Energetic

Is it mostly still affects because after you stay up late, it definitely means that you need a longer time the next day to replenish your own sleep, and it is possible to waste a little bit of the next day's energy, such as maybe the next day if you don't stay up late, you can put more energy into the things that you like to do. (p21)

Discomfort

He was in the normal course of treatment and then he had a bleed, a gastrointestinal bleed, and then at first he was feeling more nauseous (P16)

Pain

He would have some cancer pains or just be very unwell (p16)

Appetite

For example, if they don't want to eat, they don't want to eat even if you bring them food (p22)

Appearance

Not enough depth of sleep, not enough sleep, difficulty in falling asleep, very dark face, and then very anxious, would have stronger sort of ruminations, and then would have overly large and overly exaggerated reactions to things around them. (p19)

# Relationship

Betray

But suddenly that person started a new section of life after utilizing him, that is, he himself felt stabbed, betrayed, and then also paid a lot of economic, and then time and energy are all paid, but nothing gained, or the child father will feel a big blow, this kind of. (p15)

Support

Spiritual pillars, first of all the first to economic-oriented, as if people say that there is no money no money, there is a disease to spend a lot of money, he took the initiative to pay the bill and reach out to take is different (P12)

Get on with people

And then but you just mentioned that he is having problems with social functioning because you said that he is not shutting himself up inside his own world, which means that he is not going to be able to communicate and interact with other people. (p14)

Socialization

Psychologically he may be immersed in the virtual world of the game and ignore the reality of interpersonal interaction, he may be to him for his interpersonal interaction of a model of the network of virtual interaction has replaced the reality of interpersonal interaction, the network of virtual interaction and the reality of interpersonal interaction. It should be a kind of two different modes of education, but if a disproportionate share of one replaces the other, then it becomes more and more closed. Can't think of a good one, (p24)

Judgement

Losing the courage to live, including for example, others will discriminate against you, and even the people around you are the people who are closer to you, the original may be very good, but now it has changed for a long time, resulting in the people around you will also discriminate against you, in addition to the life can not see the hope, it will be felt that after how many years or this, there is not much too much of this hope. (P17)

Sense of belonging

Rich in the mountains with distant relatives, poor in the city without a single question (P9)

Loneliness

He said they are all dead, his brother is dead, his wife is dead, his son died last year, and I feel particularly sorry for the old man, all alone by himself leading a mentally disabled brother (p14)

Burden

And to the extent that it is in my power to do so, I can carry out the care of myself alone, it should not be alone, for example, if I feel that I myself for example, although I have mobility problems, I feel that my own need for occasional help from other people is not a burden to other people. (p17)

Interpersonal relationships

Right, also can not afford to go, at the time of leaving, he held my father's hand and said can not afford to do so, but he is also considered lucky, go to the hospital as if a stay a night, the next day there is nothing no pain torture, and there is nothing to go very calmly, there is no torment, is calmly gone, life is like this, no drag before going, is no pain and drag the family, can be counted, and also nothing Go also peace of mind, so that we I and my brother and sister and lover all at his bedside, go okay, nothing, people can see, basically. (P13)

# Activity

Enjoyable or meaningful activities/role

We actually have higher spiritual needs, for example, I am not just going to do this thing, is to want to do it well, and then in their own interests on top of their own ideas and creativity, if it is such a repetitive hard labor, then it will be mechanical labor, it will suppress our brain's active area of activity, and then it will also make us if we waste too much time in the work, we won't end up having a hard time reaching the goals that each of us wants to reach on a spiritual level. (P1)

Physical function

The basics of living and breathing, eating, sleeping and all that. And then to take it up a little bit more is recreational or sports reading, and some more in-depth learning. (p1)

Daily activity

There's nothing else that, anyway it's at home now helping the girl to do some cooking, taking the kids to school, and in other things there's nothing else, sometimes going up to the park for a while, and we older people don't have anything else in mind right now. (P4)

Self-care

First of all, she can't take care of herself, she's sick, she's paralyzed in bed, she can't get what she wants to eat, she can't get what she wants to drink, she has to be taken care of, and she also has to take care of her own emotions, and most of all, she has to take care of her own emotions, and most of all, she has to take care of her own emotions (P12)

Diet

The most important thing is diet, such as myself after I got sick, after surgery, ginger, onion and garlic can not eat, frying and spicy can not eat, such as my husband cooked some of my own and their own appetite, and do not dare to say, simply their own sick also cook their own, because if you say ehhh I can not eat onions and can not eat garlic, so to him and what. I am able to move when I will cook, even if I am not comfortable, I can cook when I will cook, first of all, there is this mood, knowing that I can not eat this way to cook, is not want me to die. These are unavoidable, dietary aspects (p12)

Mobility

For example, he has bad legs and he can't run and jump like us. (P14)

Vision

The ability to walk and travel. And for example if you have problems with your eyes and you can't see, and if you have problems with your lower limbs or anything else, or even if you have to stay in bed (p1)

Verbal expression

Then he had no way of recognizing the people around him and then slurred his speech, and he would speak with words that didn't come out right kind of feeling (P19)

Sleep

After watching TV and using the phone for too long, sometimes I feel very tired but can't sleep, which affects my ability to fall asleep.(p29)

# Mindset

Life attitude

Quality of life means that your life is your own, and you need to value it. You should not treat your life as a joke. (p9)

Adjust mindset

Depending on the severity of the illness, if the disease is severe and beyond one's ability to cope, they are likely to feel very pessimistic and despondent. (p10)

After falling ill, my mood definitely affects my condition. However, once I let go and stop worrying about other things, everything seems fine. Once I change my mindset, I no longer mind. For example, when I initially felt unwell, I found fault with everything my husband cooked. But later, when I changed my perspective, it didn't bother me anymore. (p12)

Positive/negative energy

He tends to blame others, attributing his own miserable life to his relatives and siblings. He believes it's their fault for not helping him. He feels they should assist him with his tasks but don't, and as a result, he often blames others and sometimes even resorts to scolding them. (p15)
